# A real-world retrospective study of safety, efficacy, compliance and cost of combination treatment with rush immunotherapy plus one dose of pretreatment anti-IgE in Chinese children with respiratory allergies

**DOI:** 10.3389/fimmu.2022.1024319

**Published:** 2022-10-04

**Authors:** Pingping Zhang, Sainan Bian, Xibin Wang, Zhuanggui Chen, Lifen Yang, Feng Xiao, Kai Guan

**Affiliations:** ^1^ Department of Pediatrics, Third Affiliated Hospital of Sun Yat-sen University, Guangzhou, China; ^2^ Department of Allergy, Third Affiliated Hospital, Sun Yat-Sen University, Guangzhou, China; ^3^ Department of Allergy, Peking Union Medical College Hospital, Peking Union Medical College, Chinese Academy of Medical Sciences, Beijing, China; ^4^ Department of Stomatology, Third Affiliated Hospital of Sun Yat-sen University, Guangzhou, China

**Keywords:** rush immunotherapy, anti-IgE, mite allergen, respiratory allergies, children

## Abstract

**Background:**

The efficacy of allergen immunotherapy (AIT) in treating pediatric allergy has been clearly demonstrated, however, many patients hesitate to initiate AIT due to weekly hospital visits during the 3-4 months up-dosing phase. Meanwhile, rush immunotherapy (RIT) shortens the duration of the up-dosing phase to 7 days. However, considering that patients receiving RIT are exposed to the allergens during a much shorter period of time and thus may be at a greater risk of systemic reactions, RIT is currently underused, especially in children. This study investigated the utility of combination treatment with RIT plus 1 dose of pretreatment anti-IgE in children with respiratory allergies.

**Methods:**

In this retrospective study, we reviewed records of children with allergic rhinitis (AR) and/or allergic asthma (AA) sensitized to dust mite allergens receiving RIT+1 dose of pretreatment anti-IgE (the RIT group) or conventional immunotherapy (the CIT group) at our hospital from January 2020 to March 2021. Data such as visual analogue scale (VAS) scores, comprehensive symptom and medication score (CSMS), allergy blood test results, adverse reactions, compliance and cost were collected and analyzed.

**Results:**

40 patients in the RIT group and 81 patients in the CIT group were included in this study. Both treatments were well tolerated and patients in the 2 treatment groups had comparable local and systemic reactions. Compared to CIT, RIT + anti-IgE combination led to significantly faster symptomatic improvement as demonstrated by significantly decreased VAS and CSMS starting as early as 1 month after AIT initiation (*P*<0.05). Nobody dropped out in the RIT group during the 1 year follow-up, while 11 out of 81 patients in the CIT group dropped out (loss rate 13.5%). Thus, the RIT group had a significantly higher compliance rate than the CIT group (P<0.05). Finally, the 2 treatment regimens had comparable cost per patient per injection (*P*> 0.05).

**Conclusions:**

RIT + 1 dose of pretreatment anti-IgE combination has practical advantages over CIT, including comparable safety, better compliance, and probably a faster onset of clinical efficacy at no additional cost, so it can be an useful regimen for the treatment of Chinese children with respiratory allergies.

## Introduction

Since its first introduction by Noon in the early 1900s (1), allergen immunotherapy (AIT) has been used to treat allergic diseases and its efficacy has been clearly demonstrated, particularly for treating respiratory allergies (allergic rhinitis [AR] and allergic asthma [AA]) (2). However, although the efficacy of AIT has been repeatedly reported by numerous clinical trials and meta-analyses, it remains underused and was used in less than 10% of patients with AR and/or AA worldwide (2). In addition, the proportion of patients completing the 3-year AIT treatment course was as low as 30% of those who initiated the treatment (3). During the initial 3 to 4-month build-up (up-dosing) phase of the conventional subcutaneous AIT, patients need to make weekly hospital visits and this is a major contributor to patients’ reluctance to initiate AIT. Addressing this key factor for low patient compliance is needed to optimize an immunotherapy strategy that could improve patients’ convenience and experience (4). Rush subcutaneous immunotherapy (RIT) with markedly shorter duration of initial build-up phase was introduced in an effort to improve patient convenience and experience, especially during the Coronavirus (COVID-19) pandemic, when minimizing the need for patients to make hospital visits was advised to reduce the risk of spreading the COVID infections. During recent years, there have been several studies on RIT (4–7). Some reports (2, 8) found that RIT had a good safety and efficacy and was well tolerated, especially when administered in combination with anti-IgE (Omalizumab) (9–11),pretreatment of Omalizumab could quickly lessen patients’ adverse reactions to RIT. However, data on combination RIT in pediatric patients are scarce. In the current retrospective real-world study, we compared the efficacy, safety, patients compliance, and cost of RIT + 1 dose of pretreatment anti-IgE with conventional AIT in pediatric patients with mites-induced allergies treated at our center during recent years.

## Materials and methods

### Patients

This is a single-center, real world, retrospective study performed at the Third Affiliated Hospital of Sun Yat-sen University, Guangzhou, China. This study included all of the patients aged 5 to 16 years with mite-induced AR and/or AA treated with RIT + 1 dose of pretreatment anti-IgE (Omalizumab) or conventional AIT at our hospital from January 2020 to March 2021. Patients with severe asthma were excluded. Diagnosis of asthma and its severity were periodically assessed and reassessed for each patient according to the Global Initiative for Asthma (GINA) Guidelines (7). AR were diagnosed and managed according to the British Society for Allergy and Clinical Immunology (BSACI) guideline (12). All patients took allergen tests to confirm that mites were the only allergens, or, in the case of patients with multiple sensitization, the main allergens of 2 or 3 types of allergens. A patient’s allergy to mites was confirmed when the skin prick test was positive (≥++), or his/her serum level of anti-mite specific IgE (sIgE) was ≥ ++. Finally, all patients took pulmonary function tests to determine whether they had complication(s) of asthma.

Patients were assigned to 2 treatment groups based on the treatment regimen they received: the RIT group received RIT in combination with 1 dose of pretreatment anti-IgE (Omalizumab) and the CIT group received conventional AIT (CIT). Patients in the RIT group were hospitalized and administered with one dose of anti-IgE (Omalizumab) before RIT.

This study was approved by the Ethics Committee of our hospital (The Third Affiliated Hospital of Sun Yat-sen University, Guangzhou, China). All patients provided written informed consent before starting treatment. In addition, all of them also gave written informed consent for publication of this study.

### Treatment

To ensure safety, all patents were treated with oral antihistamines before each subcutaneous injection of the allergen shot in the morning, and they were monitored for at least 1 hour after each injection. Mite preparations containing a mixture (1:1) of dermatophagoides pteronyssinus (DP) and dermatophagoides farinae (DF) extracts ^®^ (Allergopharma Joachim Ganzer KG, Reinbek, Germany) were used as allergen shots for subcutaneous immunotherapy in this study. The highest concentration of the mite allergen shot used had an allergenic activity of 5,000 TU/mL. The first maintenance dose was administered on day 7 for patients in the RIT group and on week 14 for patients in the CIT group. After the first maintenance dose, patients entered maintenance phase and were maintained at a dose of 1.0 mL (5,000TU/mL). Detailed RIT and CIT schedules were described in **Table 1**.

**Table 1:** RIT and CIT schedule.

Time	RIT					CIT				
	Stage	Injection No.	Number of vials	Volume(ml)	Dose (TU)	Stage	Injection No.	Number of vials	Volume(ml)	Dose (TU)
									
D1	Up-dosing phrase	1	1	0.1	5	Up-dosing phrase	1	1	0.1	5
		2	1	0.2	10					
D2		3	1	0.4	20					
		4	1	0.8	40					
D3		5	2	0.1	50					
		6	2	0.2	100					
D4		7	2	0.4	200					
		8	2	0.8	400					
D5		9	3	0.1	500					
		10	3	0.2	1000					
D6		11	3	0.4	2000					
		12	3	0.6	3000					
D7		13	3	1	5000					
W2	Maintenance phrase						2	1	0.2	10
W3							3	1	0.4	20
W4							4	1	0.8	40
W5		14	3	1	5000		5	2	0.1	50
W6							6	2	0.2	100
W7							7	2	0.4	200
W8							8	2	0.8	400
W9							9	3	0.1	500
W10		15	3	1	5000		10	3	0.2	1000
W11							11	3	0.4	2000
W12							12	3	0.6	3000
W13							13	3	0.8	4000
W14							14	3	1	5000
W15		16	3	1	5000	Maintenance phrase				
W16										
W17										
W18							15	3	1	5000

RIT, rush subcutaneous immunotherapy+1 dose of anti-IgE combination; CIT, conventional allergen immunotherapy; D, day; W, week.

Compared to patients in the CIT group, patients in the RIT group were exposed to a large amount of allergens during a much shorter period of up-dosing phase, therefore, the 7-day up-dosing phase of RIT was conducted in a hospital setting to monitor and timely treat the possible systemic reactions. Patients in the CIT groups received their treatment in our outpatient department. Patients in both the RIT and CIT groups were administered with oral antihistamines according to instruction (Loratadine tablets, 5mg for bodyweight < 30 kg,10mg for≥30 kg) at least 1 hour before each injection of mite allergen shot. In addition, all patients in the RIT group were given a shot of anti-IgE (150mg Omalizumab, Novartis Pharma Stein AG, Stein, Switzerland) subcutaneously at least 1 hour before starting AIT. In addition, peak expiratory flow (PEF) was measured 3 times both before and after each injection using a peak flow meter. If a patient’s mean PEF was higher than 70% but ≤80% of the predicted normal value (5), he/she would be given symptomatic treatment and injections of allergen shot would be given under close observation. If a patient’s mean PEF value was ≤70% of the predicted normal value (5), the patient would stop receiving injections and be given appropriate treatment. The injection would be resumed only after the patient’s PEF value returned to normal (5). All patients were observed at least for 1 hour after the injection. The detailed information about regimen was listed in **Table 1**.

### Treatment efficacy, safety, compliance and cost

Visual analogue scale (VAS) and comprehensive symptom and medication score (CSMS) were used to evaluate treatment efficacy in both groups (13). All patients took VAS and CSMS for 4 times: Week 0 (before treatment, W0), Week 5 (1 month after the start of AIT, injection of first maintenance phase dose for patients receiving RIT, W5), Week 26 (6 months after the start of AIT) and Week 52. Furthermore, peripheral blood samples were collected at Week 0, Week 26 and Week 52 for the purpose of serological tests such as total immunoglobulin G4 (tIgG4), total immunoglobulin E (tIgE), specific IgE (sIgE) (quantitative, mean value of sIgE against Dermatophagoides pteronyssinus [d1] and Dermatophagoides farina [d2]), and eosinophil (EOS) counts. tIgE and sIgE were measured with ImmunoCAP assay,tIgG4 with ELISA kit (Binding Site company, Birmingham, UK) and allergic protein components sIgE (Derf and Derp mite allergen chip method, Thermo Fisher Scientific,Massachusetts, USA), skin prick diagnostic kit (Wolwo Bio-Pharmaceutical Co., Ltd. Huzhou, Zhejiang, China) were also used in this study.

Safety of the treatments was evaluated using rates of local adverse reaction (LR) and systemic adverse reaction (SR), and the types of LR and SR experienced by the patients recorded in the medical record system were retrospectively analyzed. The severity of SR associated with AIT was classified according to the World Allergy Organization grade system (14). When a patient experienced a SR, emergency rescue procedures were activated immediately. Besides, all of the patients were followed up for 1 year.

Patient compliance was evaluated using the drop-off rate, if a patient did not come back for injection for more than 4 weeks during the up-dosing phase or more than 12 weeks during the maintenance phase, he/she was regarded as a drop-out.

The mean cost of each injection (transportation expense, doctor’s service fee, injection fee and observation fee) for each patient was also calculated and analyzed.

### Statistical analysis

All statistical analyses were performed using SPSS version 25.0 (IBM Corp, Armonk, NY, USA).Continuous variables were described as means ± standard deviations (SD). *T*-test was used to compare inter-group continuous variables that had a normal distribution and homogeneous variances, and Wilcoxon test was used to compare inter-group continuous variables that did not meet the conditions for *t*-test. Categorical variables were expressed as frequency or percentage and chi-square test was used to compare categorical variables between the 2 treatment groups. Fisher’s exact test was used for categorical variables that did not meet the condition of chi-square test. A P value < 0.05 was considered statistically significant.

## Results

### Patient demographics and baseline characteristics

A total of 121 patients were included in the study. Among them, 40 patients were in the RIT group, including 26 males and 14 females. The RIT group included 32 cases of AR, 6 cases of AA and 2 cases of AR+AA. As for the CIT group, there were 81 patients, including 59 males and 22 females. The CIT group included 64 cases of AR, 6 cases of AA and 11 cases of AR+AA. Patient demographics and baseline clinical characteristics were described in **Table 2**. The 2 groups had comparable demographics and baseline characteristics including comparable VAS and CSMS scores (*p*>0.05) (**Table 2**).

**Table 2:** Patient demographics and baseline characteristics.

Variables	RIT (n=40)	CIT (n=81)	*t/x^2^* value	*p* value
Gender (Male/Female), n/n	26/14	59/22	1.791	0.181
Age (year)	9.85 ± 3.37	8.4 ± 2.40	1.653	0.103
Parents’ education degree Bachelor or above^△^, n(%)	26 (65.00%)	56 (69.14%)	0.753	0.382
Family income (RMB)			0.524	0.471
≤25000/month,	32 (80.00%)	58 (71.60%)		
>25000/month,	8 (20.00%)	23 (28.40%)		
Diagnosis			6.982	0.065
AR	32 (80.00%)	64 (79.01%)		
AA,	6 (15.00%)	6 (7.41%)		
AR and AA	2 (5.00%)	11 (13.58%)		
Family history Allergic diseases			2.215	0.142
Yes	12 (30.00%)	36 (44.44%)		
No	28 (70.00%)	45 (55.56%)		
Multiple sensitization^✩^			0.168	0.691
Yes	6 (15.00%)	10 (12.35%)		
No	34 (85.00%)	71 (87.65%)		
Clinical assessment				
VAS score	5.30 ± 0.97	5.37 ± 1.03	0.345	0.729
CSMS score	3.24 ± 0.53	3.32 ± 0.85	0.563	0.575

Continuous variables were expressed as means ± SD while categorical variables were expressed as N (%) unless otherwise indicated.

^△^at least one parent’ education degree was bachelor or above.

✩patients with other allergens besides allergy to mites.

RIT, rush subcutaneous immunotherapy+1 dose of anti-IgE combination; CIT, conventional allergen immunotherapy; AR, allergic rhinitis;, AA, allergic asthma;, VAS, visual analogue scale; CSMS, comprehensive symptom and medication score.

### Safety - adverse reactions

During the first year after initiating AIT, 23 LR events (2.13% of the injections) were observed in 6 (6/40, 15%) patients in the RIT group, and 27 LR events (1.54% of the injections) were observed in 9 (9/70, 12.86%) patients in the CIT group. All LR events in the two treatment groups were immediate and unrelated to the patients’ allergic disease (wheal diameters <5 cm). In the RIT group, 6 SR events (0.56% of the injections, 4 grade I and 2 grade II) were observed in 5 (5/40, 12.5%) patients. As for the CIT group, 8 SR events (0.46% of the injections, 7 grade I and 1 grade II) were observed in 6 (8/70, 8.57%) patients (**Table 3**). There was no significant difference in rate of adverse reactions between the two treatment groups (*p* > 0.05) (**Table 3**).

**Table 3:** Adverse reactions in the RIT and CIT groups.

Adverse reactions	RIT (n=40)		CIT (n=70)	
	Events, n (% of total injections)	Patients, n (%)	Events, n (% of total injections)	Patients, n (%)
LR				
	23 (2.13%)	6 (15.00%)	27 (1.54%)	9 (12.86%)
SR				
Grade I	4 (0.37%)	3 (7.50%)	7 (0.40%)	5 (7.14%)
Grade II	2(0.19%)	2(5.00%)	1(0.06%)	1(1.43%)
Grade III	0	0	0	0
Grade IV	0	0	0	0

No significant differences in rates of LRs and SRs between the 2 groups (p > 0.05) RIT, rush subcutaneous immunotherapy+1 dose of anti-IgE combination; CIT, conventional allergen immunotherapy; LR, local reaction; SR, systemic reaction.

### Treatment efficacy - VAS, CSMS, sIgE, tIgE, EOS and tIgG4

**Figure 1:**
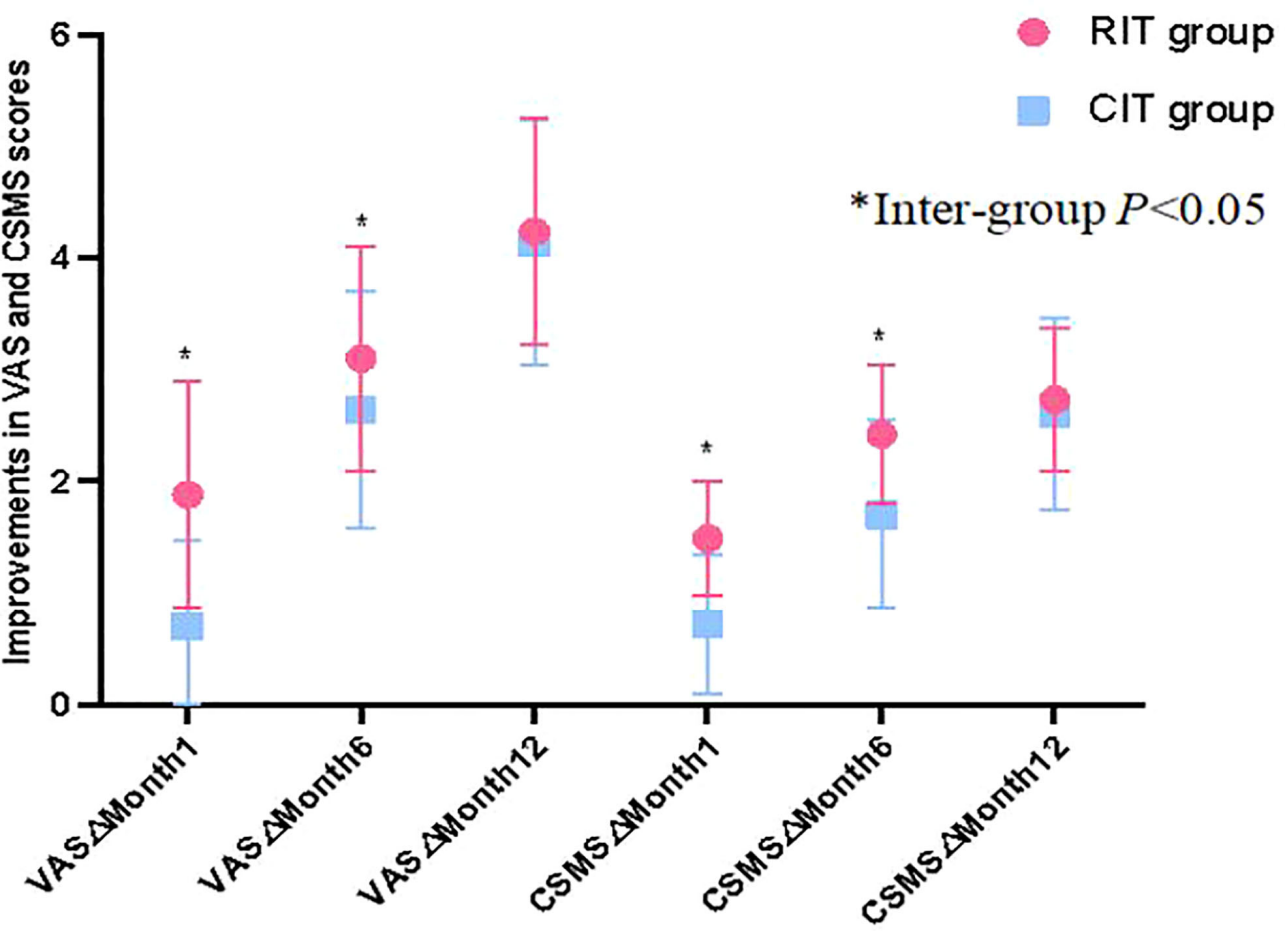
The comparision of clinical improvement. Patients in the RIT group had significantly greater VAS and CSMS improvements (reductions) from baseline than patients in the CIT group at months 1 and 6, although differences in VAS and CSMS improvement between the 2 groups became insignificant at Month 12.

VAS and CSMS scores for patients in both groups had a downward trend as treatment continued (**Table 4**). At Month 1, only patients in the RIT group had significantly reduced VAS and CSMS compared to baseline (*t*=2.971,7.705; *p*=0.004,0.001 respectively, *p* both<0.05),while improvement in VAS and CSMS score for patients in the CIT group was insignificant. In addition, compared to patients in the CIT group, patients in the RIT group had a significantly greater reduction in VAS and CSMS scores than the baseline at months 1 and 6 (month 1 *t*= -6.298,-6.346;*p* =0.011,0.000 respectively;month 6 *t*=-2.108,-4.490;*p*=0.038,0.000 respectively,*p* all<0.05), although differences in VAS and CSMS score reduction became insignificant between the 2 groups at month 12 (both *p*>0.05) (**Table 4**), suggesting that patients in the RIT group had faster symptomatic improvement than patients in the CIT group (**Table 4**, **Figure 1**).

**Table 4:** VAS and CSMS improvements for patients in the RIT and CIT groups.

	RIT group (n=40)			CIT group (n=70)		
	△Month1	△Month 6	△Month 12	△Month1	△Month 6	△Month 12
VAS	1.88 ± 1.02^*^	3.10 ± 1.01^*^	4.24 ± 1.02	0.70 ± 0.77	2.64 ± 1.06	4.14 ± 1.09
CSMS	1.49 ± 0.51^*^	2.42 ± 0.63^*^	2.73 ± 0.64	0.72 ± 0.62	1.70 ± 0.84	2.61 ± 0.86

Values were expressed as means ± standard deviations.

△means the improvement of the symptoms, that is, baseline score minus the score of the corresponding month.

*Significant difference in score reduction between the 2 treatment groups (p<0.05).

RIT, rush subcutaneous immunotherapy+1 dose of anti-IgE combination; CIT, conventional allergen immunotherapy.

Patients in RIT and CIT treatment groups had significantly increased tIgE (*t*= -5.140,-2.983;*p*=0.000,0.004 respectively) and sIgE levels(*t*=-4.087,-2.673;*p*=0.001,p=0.000 respectively) compared to baseline at month 6 (*p* all<0.05) which then decreased back to a level that is not significantly different from baseline at month 12 (*p*>0.05) (**Table 5**). In addition, EOS counts decreased significantly at months 6 and 12 from baseline in RIT (*t*=2.042,2.630; *p*= 0.045,0.010 respectively) and CIT groups (t=2.534,3.332; *p*=0.013,0.001 respectively, *p* all<0.05), while tIgG4 level in neither group had significant change over the 1 year after AIT initiation (*p*>0.05) (**Table 5**). Finally, the differences in levels of tIgE, sIgE, tIgG4 and EOS between the RIT and CIT groups at baseline, months 6 and 12 were not statistically significant (*p*>0.05) (**Table 5**).

**Table 5:** Hematological parameters for patients in the RIT and CIT groups.

	RIT (n=40)			CIT (n=70)		
	Baseline	Month 6	Month 12	Baseline	Month 6	Month 12
sIgE (kU/L)	76.92 ± 46.53	198.25 ± 91.49^*^	91.85 ± 61.24	80.77 ± 34.03	186.80 ± 112.35^*^	111.55 ± 103.14
tIgE (kU/L)	468.56 ± 317.91	1093.53 ± 700.21^*^	550.13 ± 365.00	563.86 ± 543.63	912.50 ± 633.20^*^	541.31 ± 272.67
EOS (10^9/L)	0.48 ± 0.33	0.34 ± 0.25^*^	0.32 ± 0.17^*^	0.56 ± 0.44	0.38 ± 0.24^*^	0.34 ± 0.16*
tIgG4 (g/L)	0.75 ± 0.41	0.79 ± 0.41	0.86 ± 0.42	0.98 ± 0.94	1.01 ± 0.90	1.10 ± 0.76

Values were expressed as means ± standard deviations.

*Significant change from baseline (p<0.05).

No statistically significant difference in levels of sIgE, tIgE, EOS or tIgG4 between the 2 groups was found at any time point (p>0.05).

RIT, rush subcutaneous immunotherapy+1 dose of anti-IgE combination; CIT, conventional allergen immunotherapy; EOS, eosinophils.

### Compliance and cost

**Figure 2:**
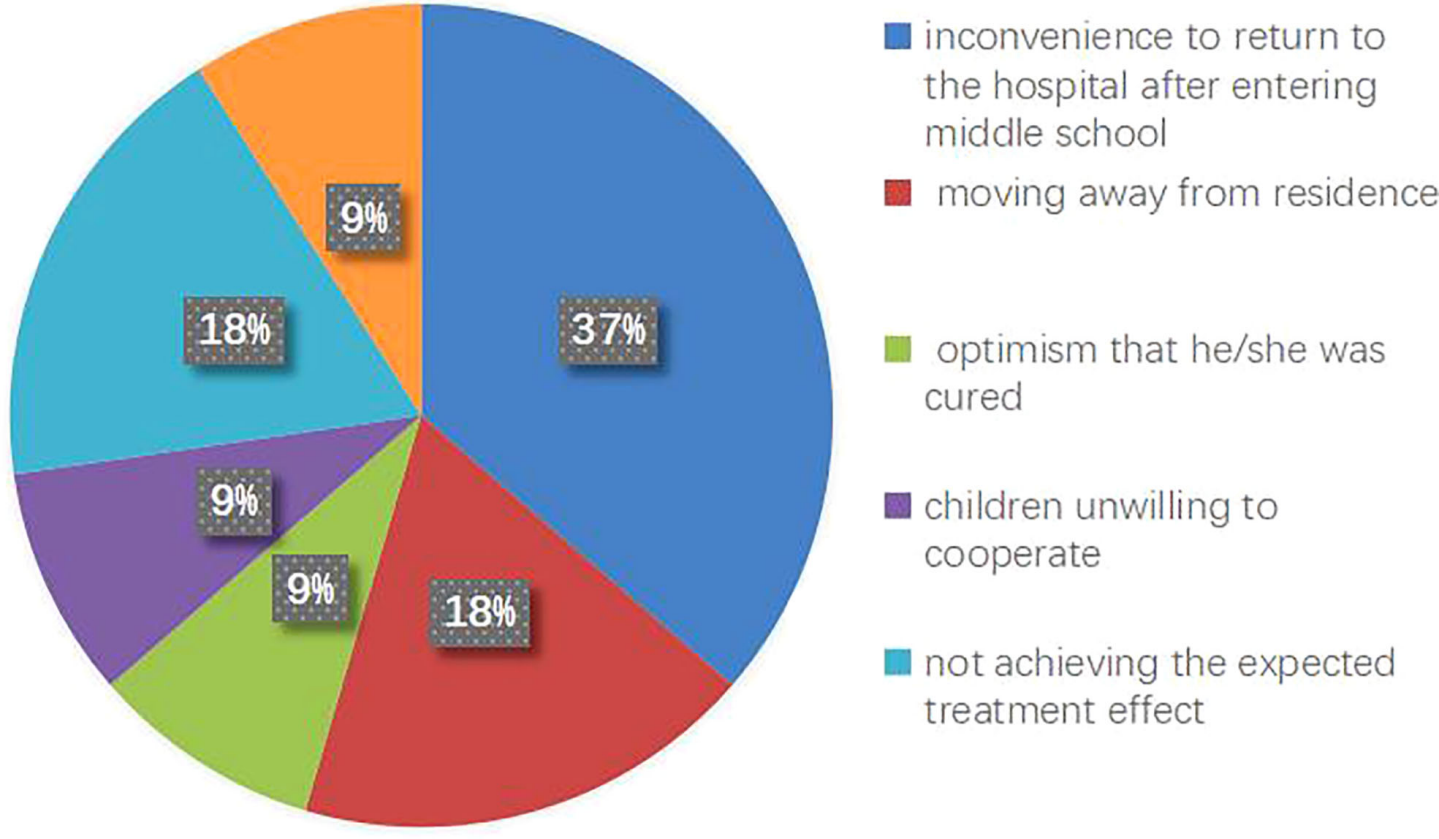
Reasons for patient drop-out in the CIT group.

A total of 121 patients were included in this study, and they were followed up for 1 year. Nobody dropped out in the RIT group, while 11 patients dropped out in the CIT group (dropout rate 13.5%). Among the 11 patients, 8 were male (8/59 male patients, 13.56%) and 3 were female (3/22 female patients, 13.63%). 7 of the 11 patients dropped out during the up-dosing phase and the remaining 4 dropped out during the maintenance phase. There were significant differences in the compliance between the two treatment groups (*x^2 =^* 5.975, *p*=0.015). The reasons for the dropout were as follows: 4 patients found it inconvenient to return to the hospital after they entered middle school, 2 moved away from their residence, 1 felt that he/she was cured, 1 child was unwilling to cooperate anymore, 2 were unwilling to continue as they felt that the treatment had not achieved the expected treatment effect, and 1 was lost to follow-up (**Table 6**, **Figure 2**).

**Table 6:** Patient compliance in the RIT and CIT groups.

	RIT (n=40)	CIT (n=81)	*x^2^* value	*p* value
Number of drop-outs/rate, n/%	0/0.00%	11/13.58%	5.975	0.015

RIT, rush subcutaneous immunotherapy+1 dose of anti-IgE combination; CIT, conventional allergen immunotherapy.

The treatment cost of each patient included transportation expenses, drug and doctor’s service fees, injection fees and observation fees, and for patients in the RIT groups, 7-day hospitalization expenses. The mean cost for each patient to receive 1 injection was RMB 325.85 ± 55.21 in the RIT group and RMB 313.93 ± 71.19 in the CIT group, and there was no significant difference between the two groups (*t*=0.837, *p* > 0.05).(**Table 7**).

**Table 7:** Treatment cost for patients in the RIT and CIT groups.

	RIT (n=40)	CIT (n=70)	t value	*p* value
Mean cost for each patient to receive 1 injection (RMB), means ± SD	325.85 ± 55.21	313.93 ± 71.19	0.837	0.330

RIT, rush subcutaneous immunotherapy+1 dose of anti-IgE combination; CIT, conventional allergen immunotherapy; SD, standard deviation.

## Discussion

Recent studies have confirmed the clinical efficacy of CIT in controlling allergy symptoms and reducing the need for medication, as well as in preventing allergic patients from developing new allergic sensitization and preventing asthma development (4, 15–17), however, it is still underused in clinical practice (2, 6, 18), mostly due to the inconvenience associated with weekly hospital visits during the 3-4 months initial build-up phase. In recent years, RIT with its much shortened up-dosing phase has achieved certain breakthroughs. The 2015 International Consensus on AIT (2) recommended RIT as an optimized and optional desensitization regimen, and a number of studies have also demonstrated its effectiveness and safety (19–23). However, most of these studies focused on patients with allergies to allergens such as pollen, grass and venom (19–23), and studies on pediatric patients have been slacking (19). Our previous research (24)found that dust mites were the main allergens in southern China, and that more than 85% of Chinese patients with respiratory allergies were allergic to mite allergens. To assess RIT vs CIT in children with respiratory allergies to mite allergens, the main allergen in southern China, in this retrospective study, we assessed efficacy, safety, compliance and cost of RIT+1 dose of pretreatment anti-IgE combination treatment and CIT in treating Chinese children with respiratory allergies to mite allergen extracts. Such a study could help pediatricians choose a proper AIT regimen for children with allergies to a common allergen in Southern China.

It is necessary to consider safety of any immunotherapy regimens. In our study, no life-threatening systemic reaction or fatal event was observed in any patients, and all of the observed LRs were mild and well tolerated. The incident rates of LRs and SRs observed in the 2 treatment groups had no significant difference, suggesting that, consistent with previous studies (8, 25), RIT+1 dose of pretreatment anti-IgE combination treatment and CIT have comparable safety profiles.

We also found that compared to the CIT group, the RIT group had a significantly greater symptomatic improvement (significantly greater VAS and CSMS score reductions) at months 1 and 6 after AIT initiation. Our finding was similar to those of previous studies (8, 22, 23). It suggested that patients receiving RIT + one dose of pretreatment anti-IgE could have symptomatic relief and improvement more quickly than patients receiving CIT, although the efficacy of the 2 treatments became similar 1 year after AIT initiation. Generally speaking, a faster improvement of allergic symptoms could provide more incentive to patients to overcome the inconvenience of a treatment regimen and to continue the treatment, thus improving patient compliance.

In the current study, patients were exposed to increased amount of allergens as desensitization treatment continued, and their clinical symptoms improved, however, patients in both treatment groups had increased serum tIgE and sIgE levels, suggesting that severity of a patient’s allergy symptom was not always related to his/her serum IgE level, the underlying mechanism of this observation needs to be further explored. Meanwhile, the EOS counts in both groups were significantly lower than baseline after 6 months’ treatment, such decrease seemed to accompany the improvement of the patients’ symptoms, which was consistent with commonly known pathological association between EOS and allergic diseases (26). Studies (27, 28) have shown that AIT could result in the production of blocking IgG/sIgG4 antibodies that can inhibit IgE-dependent activation, and allergen specific(sIgG4)level is related to the efficacy of AIT. However, in China, sIgG4 detection is only applied in research work at present, and has not been applied in clinical practice. In this study, the increase in tIgG4 level was insignificantly after AIT initiation, the short follow-up time and/or the possible lower sensitivity of the tIgG4 than the sIgG4 could potentially affect our results. However, our results regarding tIgG4 could remind clinicians that changes in tIgG4 often does not necessarily reflect AIT efficacy.

Admittedly, the high efficacy and good safety profile of RIT in our study may also be related to the following factors: (1) The high rates of adverse reactions of RIT observed in previous studies were mostly related to uncontrolled asthma attacks (29), while in our study, 80% of the patients receiving RIT were AR patients, with the remaining 20% being patients with AA or AA+AR; (2) The use of 1 dose of pretreatment anti-IgE (Omalizumab) before RIT may be a factor related to the initial high efficacy and safety of patients, Considering that RIT patients were exposed to a large amount of allergens in a short period of time, the 1 dose of pretreatment Omalizumab used in the RIT group might increase patients’ tolerance to the allergens, improve the efficacy and reduce the occurrence of adverse reactions in the RIT group to some extent (11). The higher efficacy of RIT patients at month 6 may be related to the fact that patients receiving RIT were exposed to a higher cumulative dose of allergen agents at that time than patients receiving CIT; (3) The RIT treatment reduced the needs for frequent hospital visits and could save patient’s time during the initial build-up phase, could improve patients’ symptoms quickly and thus patients would be more willing to continue the treatment as they become more optimistic about the treatment efficacy. As a result, the patients in the RIT group had a significantly a higher compliance than patients in the CIT group, and a higher compliance often could lead to a higher treatment efficacy.

Generally speaking, the incidence of adverse reactions in this study was low and well tolerated, combination treatment of RIT and 1 dose of pretreatment anti-IgE can be an useful alternative desensitization program, which is consistent with the recommendation of the international consensus on AIT in 2015 (2). Finally, we also compared the cost of the two treatments. Previous studies have described the use of anti-IgE (Omalizumab) for 2-6 months at the beginning of RIT (9, 26), but the cost associated with continued use of Omalizumab is burdensome especially in developing countries. In this study, we modified the regimen and only used one dose of Omalizumab, and the treatment cost of RIT+1 dose of pretreatment Omalizumab was comparable to CIT. As most of the individuals in RIT group were from out of town, the extra expense associated with the 1 dose of Omalizumab and 7-day hospitalization was offset by the reduced transportation time and cost as the weekly hospital visit associated with the 3-4 month up-dosing phase in the CIT was not needed for patients receiving RIT, this was consisted with the latest study (30).

The study has several limitations. First, it was a single-center study focused on dust mite desensitization, the applicability of RIT+1 dose of pretreatment Omalizumab needs to be further studied. In addition, the current follow-up period is only 1 year, and data on longer follow-up period is needed to fully evaluate the safety and efficacy of RIT + 1 dose of Omalizumab. Finally, this is a retrospective study, a properly randomized, controlled, prospective study with a larger sample size and longer follow-up period is needed to fully assess adverse reactions and its associated risk factors.

In conclusion, in order to improve the application of AIT in patients with allergic diseases, studies searching for a more reasonable and accessible approach of AIT are needed. RIT + one dose of pretreatment anti-IgE has several important practical advantages over CIT, such as comparable safety profile, faster symptomatic improvement, better compliance and no additional cost. Therefore, it can be a useful alternative regimen for the treatment of Chinese children with respiratory allergies.

## Data availability statement

The original contributions presented in the study are included in the article/supplementary material. Further inquiries can be directed to the corresponding authors.

## Ethics statement

The studies involving human participants were reviewed and approved by Ethics Committee of Third Affiliated Hospital of Sun Yat-sen University. Written informed consent to participate in this study was provided by the participants’ legal guardian/next of kin. Written informed consent was obtained from the individual(s), and minor(s)’ legal guardian/next of kin, for the publication of any potentially identifiable images or data included in this article.

## Author contributions

PZ and SB conceived, designed and drafted the work that led to the submission. XW acquired data. ZC played an important role in interpreting the results. LY and FX revised the manuscript. KG revised and approved the final version. All authors contributed to the article and approved the submitted version.

## Acknowledgments

We sincerely thank the support and help of our colleagues in the Department of Allergy and Pharmacy.

## Conflict of interest

The authors declare that the research was conducted in the absence of any commercial or financial relationships that could be construed as a potential conflict of interest.

## Publisher’s note

All claims expressed in this article are solely those of the authors and do not necessarily represent those of their affiliated organizations, or those of the publisher, the editors and the reviewers. Any product that may be evaluated in this article, or claim that may be made by its manufacturer, is not guaranteed or endorsed by the publisher.
